# The Influence of Prenatal Exposure to Methamphetamine on the Development of Dopaminergic Neurons in the Ventral Midbrain

**DOI:** 10.3390/ijms24065668

**Published:** 2023-03-16

**Authors:** Walaa F. Alsanie, Sherin Abdelrahman, Raed I. Felimban, Heba A. Alkhatabi, Ahmed Gaber, Ebtisam Abdulah Alosimi, Majid Alhomrani, Hamza Habeeballah, Charlotte A. E. Hauser, Abdulhakeem S. Alamri, Aiysha Althobaiti, Abdulaziz Alsharif, Ahmed S. Alzahrani, Mohammad S. Al-Ghamdi, Bassem M. Raafat, Khaled A. Alswat, Yusuf S. Althobaiti, Yousif A. Asiri

**Affiliations:** 1 Department of Clinical Laboratories Sciences, The Faculty of Applied Medical Sciences, Taif University, Taif 21944, Saudi Arabia; 2 Centre of Biomedical Sciences Research (CBSR), Deanship of Scientific Research, Taif University, Taif 21944, Saudi Arabia; 3 Laboratory for Nanomedicine, Division of Biological and Environmental Science and Engineering (BESE), King Abdullah University of Science and Technology (KAUST), Thuwal 23955, Saudi Arabia; 4 Computational Bioscience Research Center (CBRC), King Abdullah University of Science and Technology (KAUST), Thuwal 23955, Saudi Arabia; 5 Red Sea Research Center (RSRC), King Abdullah University of Science and Technology (KAUST), Thuwal 23955, Saudi Arabia; 6 Department of Medical Laboratory Sciences, Faculty of Applied Medical Sciences, King Abdulaziz University, Jeddah 21589, Saudi Arabia; 7 Center of Innovation in Personalized Medicine (CIPM), 3D Bioprinting Unit, King Abdulaziz University, Jeddah 21589, Saudi Arabia; 8 Center of Excellence in Genomic Medicine Research (CEGMR), King Abdulaziz University, Jeddah 21589, Saudi Arabia; 9 King Fahd Medical Research Centre, Hematology Research Unit, King Abdulaziz University, Jeddah 21589, Saudi Arabia; 10 Department of Biology, College of Science, Taif University, Taif 21944, Saudi Arabia; 11 Department of Medical Laboratory Technology, Faculty of Applied Medical Sciences in Rabigh, King Abdulaziz University, Jeddah 21589, Saudi Arabia; 12 Department of Radiological Sciences, College of Applied Medical Sciences, Taif University, Taif 21944, Saudi Arabia; 13 Department of Internal Medicine, School of Medicine, Taif University, Taif 21944, Saudi Arabia; 14 Department of Pharmacology and Toxicology, College of Pharmacy, Taif University, Taif 21944, Saudi Arabia; 15 Addiction and Neuroscience Research Unit, Taif University, Taif 21944, Saudi Arabia; 16 Department of Clinical Pharmacy, College of Pharmacy, Taif University, Taif 21944, Saudi Arabia

**Keywords:** methamphetamine, dopaminergic neurons, fetal neurodevelopment, ventral midbrain, embryonic neurons

## Abstract

Methamphetamine, a highly addictive central nervous system (CNS) stimulant, is used worldwide as an anorexiant and attention enhancer. Methamphetamine use during pregnancy, even at therapeutic doses, may harm fetal development. Here, we examined whether exposure to methamphetamine affects the morphogenesis and diversity of ventral midbrain dopaminergic neurons (VMDNs). The effects of methamphetamine on morphogenesis, viability, the release of mediator chemicals (such as ATP), and the expression of genes involved in neurogenesis were evaluated using VMDNs isolated from the embryos of timed-mated mice on embryonic day 12.5. We demonstrated that methamphetamine (10 µM; equivalent to its therapeutic dose) did not affect the viability and morphogenesis of VMDNs, but it reduced the ATP release negligibly. It significantly downregulated *Lmx1a*, *En1*, *Pitx3*, *Th*, *Chl1*, *Dat*, and *Drd1* but did not affect *Nurr1* or *Bdnf* expression. Our results illustrate that methamphetamine could impair VMDN differentiation by altering the expression of important neurogenesis-related genes. Overall, this study suggests that methamphetamine use may impair VMDNs in the fetus if taken during pregnancy. Therefore, it is essential to exercise strict caution for its use in expectant mothers.

## 1. Introduction

According to the United Nations Office on Drugs and Crime’s most recent estimate, approximately 14 to more than 53 million people worldwide are abusers of the addictive, illegal, narcotic methamphetamine [1]. Methamphetamine is a neurotoxic drug that can cause prolonged consequences in abusers despite its considerable popularity as a recreational drug due to its widespread availability, relative affordability, and longer euphoric effects [2,3,4]. The short-term administration of methamphetamine results in behavioral changes induced by the activation of dopaminergic systems in various parts of the brain [5]. Long-term abuse of methamphetamine can lead to neuropsychiatric adverse effects, including addiction, psychosis, and cognitive impairments [6], and it can also cause Parkinsonism [7]. Furthermore, some cognitive deficits are connected to the neurodegenerative alterations induced by methamphetamine use in human addicts [8]. Methamphetamine is one of the most popular “hard” drugs used by expectant mothers [9,10] and ranks among the most often used illicit narcotics in the Czech Republic and in eastern and middle Europe [10,11,12]. According to statistics, ~17% of female drug methamphetamine abusers in the USA used it as their major drug of choice, while ~40% used it during their pregnancy because of its anorectic effects [9]. Methamphetamine exposure during pregnancy has been shown to harm embryonic brain development because of its neurotoxicity [13]. Moreover, microglia that play crucial roles in brain development and neuronal network maintenance are affected by methamphetamine-induced neuronal impairment, which results in oxidative stress, transcription factor activation, mitochondrial metabolism malfunction, DNA damage, excitatory toxicity, apoptosis, and neuronal inflammation [13,14,15,16]. Therefore, organizations such as The National Institute on Drug Abuse in the USA have attempted to encourage research by increasing funding to understand the effects of methamphetamine use during pregnancy. However, the effects, especially the long-term consequences of prenatal exposure to methamphetamine, have not yet been explored fully [10].

Serotonin, norepinephrine, and dopamine transporters are the primary sites of action that are competitively inhibited by methamphetamine [17,18,19]. Of these three targets, increased expression of serotonin and norepinephrine transporters in the placenta has been reported in several studies [19,20]. These transporters play a primary role in maintaining the balance between amniotic fluid and circulation in the fetus [19,21]. Moreover, they have also been shown to be associated with vasoconstriction of placental vessels, thereby leading to complications such as preeclampsia [22], fetal abruption, intrauterine growth restriction, and preterm labor [19,23]. However, the effects of prenatal exposure to methamphetamine on fetal brain development during pregnancy are elusive.

Ventral midbrain dopaminergic neurons (VMDNs) play crucial roles in controlling cognitive function and motor activities. VMDNs are generated from the floor plate at the ventral midbrain during early embryonic development. During their development, the progenitors migrate from the ventricle and intermediate zones to the mantle zone, where maturation occurs. The development of VMDNs is controlled by different signaling cues [24]. These neurons are essential for regulating key functions in the brain, such as reward processing, learning movements, regulation, and motivation. Therefore, we hypothesized that assessing the effects of methamphetamine exposure on the growth of VMDNs can provide insights into its effects on brain neurogenesis. To test this hypothesis, we isolated VMDNs from embryonic ventral midbrain neurons (EVMNs) of mice and assessed the effects of methamphetamine exposure on the gene expression and morphological traits of VMDNs. Furthermore, to capture the complexity of the native brain tissue, a 3D culturing technique was employed in this study. Ultrashort self-assembling peptide-based scaffolds were used to establish a 3D in vitro VMDNs model. These peptides self-assemble into nanofibrous networks in physiological buffers without chemical or UV cross-linking, which make them suitable biomaterials for a myriad of tissue engineering applications. The peptide sequence selected in this study was successfully used in previous studies to develop 3D in vitro neuronal models [25,26].

## 2. Results

### 2.1. Methamphetamine Did Not Alter the Metabolic Activity of EVMNs

**Figure 1:**
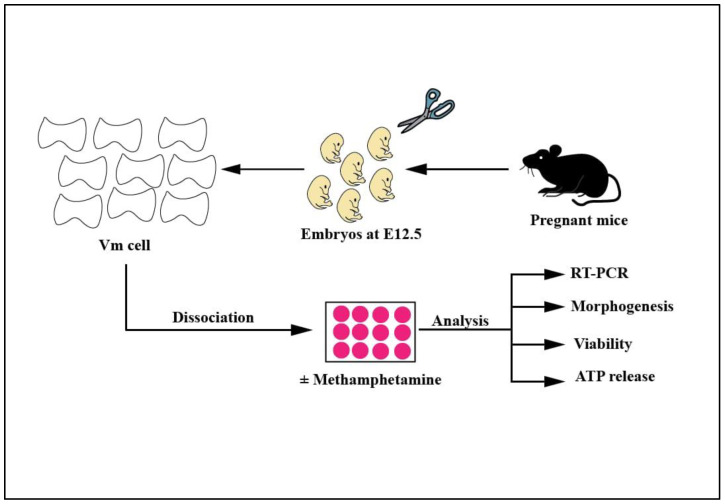
Scheme of the experimental design.

**Figure 2:**
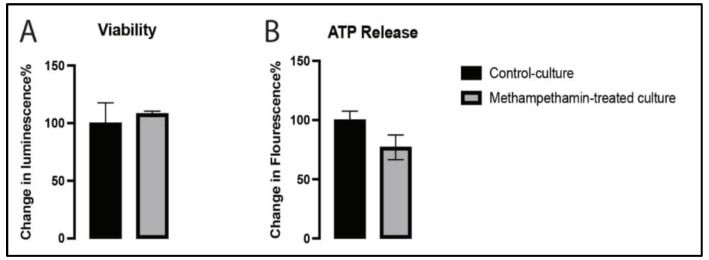
Effect of methamphetamine on the viability of embryonic ventral midbrain neurons (EVMNs) (**A**) and ATP release (**B**). The VMDNs on day three of culture were treated with 10 µM methamphetamine; control cultures were treated with phosphate-buffered saline (PBS). Data are expressed as mean ± SEM, n = three technical replicates, seven biological replicates (viability), and three biological replicates (ATP release). Data were analyzed using a *t*-test.

As shown in Figure 1, we isolated the EVMNs and cultured them for 3 days, followed by treatment with 10 µM methamphetamine, and assessed its effects on the viability of EVMNs and ATP release. Our findings showed that methamphetamine did not affect neuronal survival (*p* < 0.1173) (Figure 2A). Similarly, we observed a non-significant decrease (*p* < 0.3413) in ATP synthesis in the cultures that were exposed to methamphetamine compared to that in the control cultures (Figure 2B). These findings suggest that methamphetamine neither disrupts the mitochondrial electron transport chain (ETC) nor causes metabolic dysfunction.

### 2.2. Methamphetamine Did Not Affect the Morphogenesis of VMDNs

**Figure 3:**
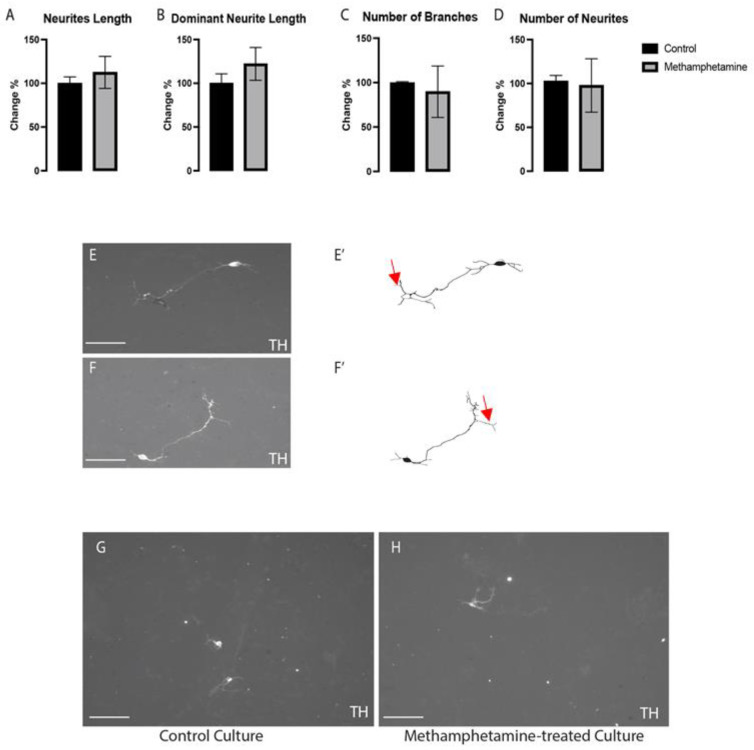
Effects of methamphetamine on neurite length (**A**), dominant neurite length (**B**), branch number (**C**), and neurite number (**D**). Representative photos and illustrations of VMDNs immunolabeled with tyrosine hydroxylase (TH) in control (**E**,**E’**) and methamphetamine-treated (**F**,**F’**) cultures. (**G**,**H**) Images show a large field of view (20×) for both groups: control and methamphetamine treated-cultures, respectively.; n = three technical replicates and four biological replicates. Data are shown as the mean ± standard error of the mean (SEM). Data were analyzed using a *t*-test. Scale bar = 50 µm (**E**,**F**). Scale bar = 10 µm (**G**,**H**). Red arrows show examples of branches.

The effects of methamphetamine on the morphogenesis of VMDNs were evaluated in immune-stained cultures with tyrosine hydroxylase (TH) and class III beta-tubulin (TUJ1). As shown in Figure 3A,B, compared to the control, methamphetamine had non-significant effects on neurite length (*p* < 0.549) and dominant neurite length (*p* < 0.344). In addition, it showed no obvious differences in the number of branches (*p* < 0.741) or neurites (*p* < 0.880) (Figure 3C,D) between methamphetamine-treated and control cultures. Representative images of immunolabeled VMDNs with TH revealed the aforementioned results (Figure 3E–H). These findings revealed that methamphetamine exposure at a dose of 10 µM did not alter the ability of neurons to differentiate.

### 2.3. Methamphetamine Did Not Change the Morphogenesis of Non-Dopaminergic Ventral Midbrain Neurons

**Figure 4:**
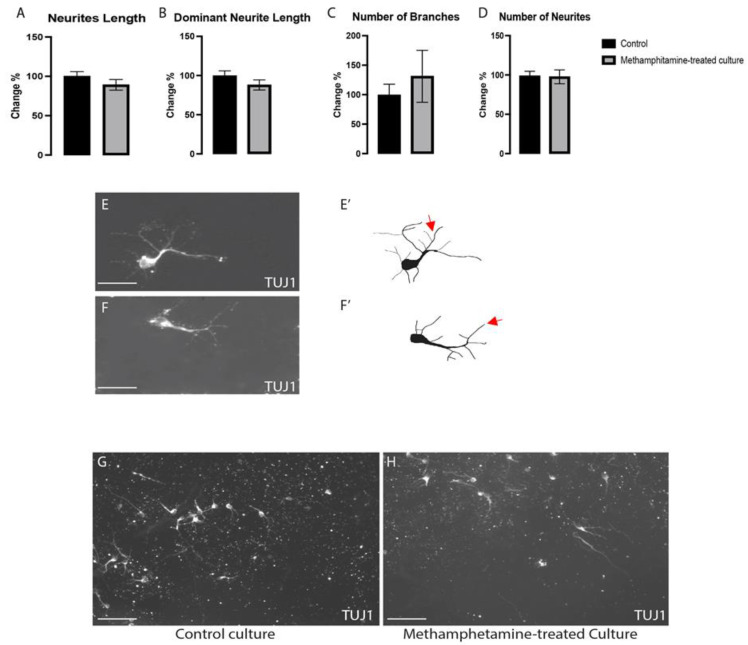
The morphogenesis of non-dopaminergic VMNs was not significantly changed upon exposure to methamphetamine (**A**–**D**). There were no discernible differences between the control (**E**,**E’**) and meth-treated (**F**,**F’**) groups in representative photos and illustrations for non-dopaminergic VMNs immunolabeled with class III beta-tubulin (TUJ1). (**G**,**H**) Images show a large field of view (20×) for both groups: control and methamphetamine-treated cultures, respectively; n = three technical replicates and four biological replicates. Data are shown as the mean ± SEM. Data were analyzed using a *t*-test. Scale bar= 50 µm (**E**,**F**). Scale bar= 10 µm (**G**,**H**). Red arrows show examples of branches.

The effects of a 10 µM dose of methamphetamine on the morphogenesis of non-dopaminergic ventral midbrain neurons (VMNs) (TH−/TUJ1+) were evaluated in labeled cultures to determine its effects on this neuronal subtype. Total neurite length (*p* < 0.285), dominant neurite length (*p* < 0.155), and the number of branches (*p* < 0.668) and neurites (*p* < 0.675) did not differ significantly between the control and methamphetamine-treated cultures (Figure 4A–H). These results suggest that 10 µM methamphetamine does not affect non-dopaminergic VMNs’ differentiation and morphogenesis.

### 2.4. Methamphetamine Altered the Expression of Dopaminergic-Related Genes in VMDNs

**Figure 5:**
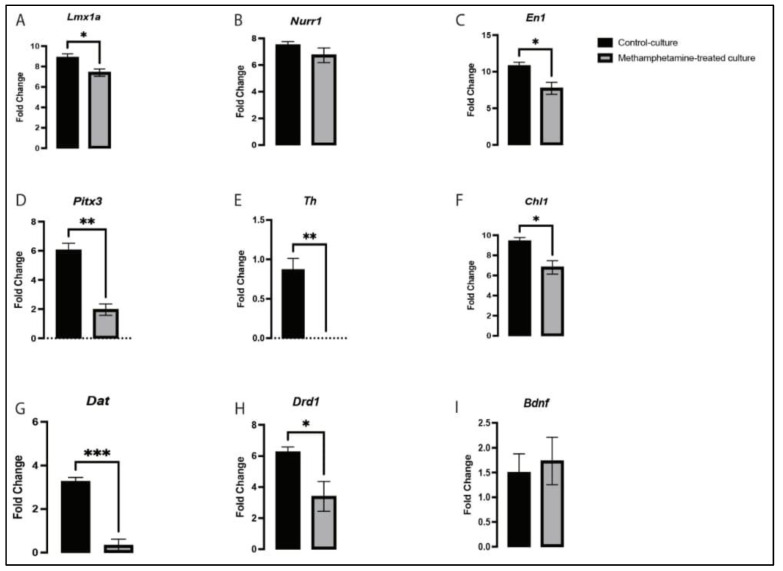
Effects of methamphetamine exposure on the expression of *Lmx1a* (**A**), *Nurr1* (**B**), *En1* (**C**), *Pitx3* (**D**), *Th* (**E**), *Chl1* (**F**), *Dat* (**G**), *Drd1* (**H**), and *Bdnf* (**I**). Data are represented as mean ± SEM; n = three technical replicates and four biological replicates; * *p* < 0.05, ** *p* < 0.01 and *** *p* < 0.001. Data were analyzed using a *t*-test.

Next, we evaluated the effects of methamphetamine on the expression of genes involved in neurogenesis. The expression of *Lmx1a* was downregulated (*p* < 0.0455) in the methamphetamine-treated cultures compared to that in the control cultures (Figure 5A). Similarly, the expression of *En1* was also significantly decreased (*p* < 0.0329) in the methamphetamine-treated cultures compared to that in the control cultures, whereas expression of *Nurr1* showed a non-significant alteration (*p* < 0.2767) (Figure 5B,C). Furthermore, methamphetamine exposure in vitro significantly reduced the expression of *Pitx3* (*p* < 0.0029) and *Th* (*p*< 0.004) (Figure 5D,E), indicating that methamphetamine affects VMDN maturation. The expression of *Chl1* was significantly downregulated (*p* < 0.0264) in the present study (Figure 5F), suggesting that neurogenesis and maturation of VMDNs were altered by methamphetamine exposure.

Furthermore, we assessed the effects of methamphetamine on downstream targets of *Nurr1*, including *Bdnf*, *Dat*, and *Drd1*, which are crucial for VMDN development and neurogenesis [24,27,28,29]. The expression of *Dat* (*p* < 0.001) and *Drd1* (*p* < 0.0472) was significantly decreased, while the expression of *Bdnf* showed a non-significant elevation (*p* < 0.7224) in response to methamphetamine treatment (Figure 5G–I). Collectively, these findings suggest that methamphetamine affects VMDN differentiation via the *Lmx1a*/*En1*/*Pitx3*/*Th*/*Chl*/*Dat*/*Drd1* pathway. It can be inferred that methamphetamine adversely affects the normal developmental course of VMDNs by altering the expression of important genes involved in neurogenesis.

## 3. Discussion

Acute administration of methamphetamine, a psychostimulant, results in behavioral changes induced by the activation of dopaminergic systems in various parts of the brain [5]. Accumulating evidence shows that exposure to methamphetamine during pregnancy causes neurotoxic effects in offspring [13]. In this study, we demonstrated that methamphetamine at 10 µM was relatively non-toxic to EVMNs and did not affect the release of ATP. Similarly, the morphogenetic analysis showed that methamphetamine (10 µM) did not alter the neurite length and number and length of branches of VMDNs and non-dopaminergic VMNs.

Nevertheless, the analysis of the expression of *Dat*, *Chl1*, Th, *En1, Drd1*, *Pitx3*, and *Lmx1a*, crucial genes for preserving regional identity in the midbrain [30], revealed that exposure to methamphetamine disrupted early neuronal development in VMDNs. Methamphetamine dramatically changed the expression of most of these genes by altering or modulating their expression. The genes *Lmx1a/b*, *Mash1*, and *Ngn2* (which regulate early DA destiny), *Wnt5a/7a* and *Netrin1* (which affect axonal plasticity), and *Wnt5a*, *Pitx3*, and *Th* (which are crucial for neuronal maturation) are important neurogenesis-related genes. However, none of these genes fully accounted for all processes involved in VMDN development [31,32,33,34]. *Lmx1a* and *b* with 64%, 83%, and 100% amino acid sequence identity in the LIM domain, LIM domain, and homeodomain domain, respectively [35], are involved in the fate and functional activities of mDA progenitors [36]. Transcription factors and genes such as *Lmx1a*, *Nurr1*, and *Mash1* allow the direct generation of VMDNs from murine and human fibroblasts without reversing to the progenitor cell stage [37]. Numerous studies have suggested that some genes, such as *Foxa2*, *Pitx3*, *Otx2*, *Nurr1*, and *En1*, are crucial for maintaining the phenotype of neurons and are associated with the early development of VMDNs [38]. According to previous studies, *Lmx1a* promotes *Nurr1*, which then activates *Th*, which is involved in VMDN neuronal development [39,40]. In an earlier work by our group and others, *Chl1* was connected to the emergence of VMDNs [40,41].

It was noted that methamphetamine exposure resulted in a substantial downregulation in the gene expression of *Dat* and *Th*. The results are consistent with those of earlier studies in which repeated high-dose amphetamine injections were administered quickly, leading to reductions in *Th* and *Dat* in the rat striatum [2,42]. Alterations in transcription factors that control the gene expression of these dopaminergic markers could also be a secondary cause of differential alterations in the levels of *Th* and *Dat* induced by methamphetamine use. Recent transcriptional investigations have also demonstrated the involvement of certain genes and elements specific to a particular lineage, including *Nurr1*, *Lmx1b*, *Pitx3*, *En1*, *Th*, and *Lmx1a*, that play key roles in the growth and preservation of the functional archetype of VMDNs [40,43,44]. Another study found that *Lmx1a* has practical utility in the child’s life after birth and is still present in mitotic residual precursors and actively specialized neurons [45]. As function-related genes have been affected by methamphetamine exposure as shown in the present study, it is critical to investigate the effects of methamphetamine on dopamine release in vitro and in vivo.

The findings of this study raise the possibility that pregnant women should not use methamphetamine because the risk of cognitive defects and neuronal harm is never minimized. However, the clinical judgment made in light of methamphetamine therapy during pregnancy may be influenced by the findings of this study. To validate the findings of this study, additional research is required to determine whether the developmental transcription factors evaluated in this study control the dopaminergic circuitry in adult brains upon exposure to other dopaminergic modulators. Additionally, it is crucial to investigate whether larger doses of methamphetamine would have similar effects on the dopaminergic-related genes as reported with the dose used in the current study.

## 4. Materials and Methods

### 4.1. Isolation of Primary Mouse Embryonic Ventral Midbrain Neurons

All animal experiments in this study (Figure 1) were carried out in compliance with international norms for the use of animals in research and were approved by the Ethics Committees of King Abdulaziz University (KAAU) and Taif University (7-CEGMR-Bioeth).

Female Swiss mice (adults) obtained from King Fahad Medical Research Center were mated with Swiss mice males at night at Animal housing at KAAU, Jeddah, SA. When a vaginal plug was visible the following morning, it was considered embryonic day (E) 0.5. The dissection of embryonic ventral midbrain has been performed as previously described [46]. Breifly, the embryos from the timed-mated mice were obtained at E12.5, and ventral midbrains (VMs) were isolated and transferred to an ice-cold L15 medium (Thermo Fisher Scientific, Waltham, MA, USA). The isthmic organizer, telencephalon, and mesencephalon boundaries were cut to separate the midbrain and cortical tissues. Tissue from the rear of the midbrain was collected to increase the number of dopaminergic cells in the culture. The separated VMs were treated with 0.05% trypsin and 0.1% DNase diluted in Ca/Mg-free Hank’s Balanced Salt Solution (HBSS) for 15 min at 37 °C. The tissues were washed three times in HBSS medium before re-incubation in N_2_ medium (comprising F12 medium, 6 mg/mL glucose, Minimum Essential Medium, 1% penicillin/streptomycin, 15 mM HEPES, 1 mM glutamine, 1 mg/mL bovine serum albumin, and 1% N_2_ supplement; Thermo Fisher Scientific). Primary neurons were prepared in vitro approximately 3 days before the study, depending on the experiments (shown in the sections below).

### 4.2. Three-Dimensional (3D) Neuronal Cell Culture and Methamphetamine Treatment

In order to recapitulate the complex brain tissue architecture, a 3D in vitro VMDN model was used in this study to assess viability, ATP release, morphogenesis, and gene expression. To this end, ultrashort self-assembling peptides proved to be promising biomaterials for the development of functional 3D neuronal models [25,26]. An Ac-Ile-Ile-Cha-Lys-NH_2_ (IIZK) tetrameric self-assembling peptide was used in this study to establish the 3D neuronal cultures as described previously [47].

The IIZK-based hydrogel was prepared in a final concentration of 2 mg/mL by dissolving the peptide powder in a volume of sterile water equivalent to half the required final volume. A peptide base was first prepared in the cell culture plates to ensure efficient 3D encapsulation of the VMDNs within the peptide scaffold. In a 96-well plate, 20 μL of the prepared peptide solution was added to each well followed by an equivalent volume of 2xDPBS to promote the hydrogel formation. The plate was then incubated at 37 °C for 5 min to ensure complete hydrogel formation. To establish the VMDN 3D construct, 10 μL of the peptide solution was added on top of the previously prepared peptide base. 6 × 10^4^ of the VMDNs in 2xDPBS were then deposited and briefly mixed with the peptide solution. The plate was then incubated again for 2–3 min. The cell culture plates were then filled with N2 media and incubated for 72 h at 37 °C and 5% CO_2_. Methamphetamine was prepared in sterile 1× PBS (Sigma-AldrichSt. Louis, MO, USA), and 10 µM methamphetamine was added to the methamphetamine-treated group. The dose was determined based on previous studies that measured the concentration of methamphetamine in blood and plasma [48,49,50,51].

### 4.3. Assessment of the Viability of EVMNs and ATP Release

We evaluated the viability of EVMNs and ATP release in response to methamphetamine treatment after three days of culture. The alamarBlue^TM^ Cell Viability Assay Reagent kit (Thermo Fisher Scientific) was used to determine the viability of the EVMNs in the control and methamphetamine-treated cultures.

The CellTiter-Glo^®^ 3D Cell Viability Assay (Promega, Madison, Wisconsin, WI, USA) was used to measure ATP release to assess the metabolic activity of the cells following the manufacturer’s instructions. Briefly, CellTiter-Glo^®^ Reagent (Promega) was added in a volume equal to that of the cell culture media in the plate and mixed by pipetting up and down 10 times to break the 3D construct comprising cells and hydrogel. Afterward, the plates were incubated for 25 min at room temperature, and the luminescent signal was recorded using a *PHERAstar* FS plate reader (BMG LabTech, Ortenberg, Germany).

### 4.4. Immunocytochemistry

VMDNs were fixed in culture using 4% paraformaldehyde after 3 days of methamphetamine treatment (and control cultures) and stored at 4°C in 1× PBS until the staining procedure. TUJ1 (1:1500; Promega) and TH (1:500; Abcam, Cambridge, UK) primary antibodies were incubated with fixed cultures overnight at room temperature in a blocking buffer comprising 5% goat serum, 0.3% Triton-X, and 0.2% sodium azide.

After removing the primary antibodies, the cells were treated with a blocking solution for 1 h at room temperature. Subsequently, the cells were incubated with goat anti-rabbit IgG H&L (Alexa Fluor^®^ 555) and anti-mouse Alexa 488 (Abcam, ab150078) secondary antibodies at 1:200 dilutions for 2 h at room temperature. The wells were then cleaned and maintained in 1× PBS and treated with DAPI (Thermo Fisher Scientific; D1306) diluted in 1× PBS for 5 min. Imaging was performed using a DMi8 inverted fluorescence microscope (Leica, Wetzlar, Germany).

### 4.5. Morphogenetic Analysis

The effects of methamphetamine on VMDN morphogenesis were evaluated in immuno-stained cultures with TH and TUJ1 following the protocol described in a previous study [41]. The length of the dominant neurites, the total number of branches, the number of neurites, and the overall length were estimated. The neurites originate from the cell body of the neurons, while the neurites that originate from other neurites are considered as branches. The analysis was performed using the Leica Application Suite X (LAS X) software (Leica), and shorter and overlapping neurites were excluded from the analysis to prevent bias. Data from cultures treated with methamphetamine were standardized to those of the control group. The data were then reported as a percentage change from the control, which was taken as 100%.

### 4.6. Quantitative PCR

The expression of important genes crucial to neuronal differentiation was examined. RNA was isolated using RNeasy Plus Universal Mini Kit (Qiagen, Hilden, Germany) following the manufacturer’s instructions. Briefly, after 3 days of methamphetamine treatment (and control cultures), the cells were homogenized using TissueLyser II (Qiagen). VMDN RNA was extracted from both methamphetamine-treated and control cultures. RNA extracted from mouse tissues other than the brain was used as a negative control. Table 1 lists the primer sequences used for the selected genes.

The raw cycle threshold (CT) data for *Gapdh* (housekeeping/reference gene) and *Nurr1*, *Pitx3*, *Drd2*, *Lmx1a*, *Th*, *En1*, and *Bdnf* (target genes) were obtained using the RT-PCR StepOne System and Data Assist software. Before analysis using the CT method, we normalized the target gene CT to the reference gene CT and those of the test sample to the control sample and calculated the differences [(CT target gene − CT reference gene); (CT test sample − CT control sample)], and finally, we calculated the relative quantification (Rq = 2 − CT) and fold change (log_2_FC) to assess the expression of the target genes under various experimental conditions. All samples from all the groups’ Rq values for each gene were compared, and *p*-values indicated significant expression of the genes.

### 4.7. Statistical Analysis

All quantitative data were expressed as mean ± standard error of the mean (SEM). GraphPad Prism v 8.1.2 was used to perform Student’s *t*-tests, and differences with a *p*-value of <0.05 were considered significant.

## 5. Conclusions

This study demonstrated the effect of methamphetamine (10 µM; equivalent to its therapeutic dose) on VMDNs, which control cognition, coordination, movement, and behavior. Using primary mouse EMVNs, we demonstrated that methamphetamine consumption during pregnancy at doses normally used therapeutically is potentially harmful to the neuronal development of the developing fetus. Exposure to methamphetamine considerably downregulated *Pitx3*, *Th*, *Lmx1a*, *Dat*, *En1*, *Chl1*, and *Drd1* expression, suggesting that methamphetamine adversely affects the normal developmental course of VMDNs by altering the expression of important genes involved in neurogenesis and affects VMDN differentiation via the *Lmx1a*/*En1*/*Pitx3*/*Th*/*Chl*/*Dat*/*Drd1* pathway. Overall, the study suggests that the clinical use of methamphetamine in expecting mothers must be exercised with strict caution, keeping these findings under consideration. However, to understand how methamphetamine influences the formation, functionality, and behavior of VMDNs, further studies are required to investigate the effects of methamphetamine on VMDNs in vivo, which may help guide clinical decisions on using this drug.

## Figures and Tables

**Table 1:** Sequences for the gene-specific primer pairs used in RT-PCR.

Gene Name	Primer Sequence 5 to 3	
*Gapdh*	Forward primer: Reverse primer:	TGA AGG TCG GAG TCA ACG GA CCA ATT GAT GAC AAG CTT CCC G
*Th*	Forward primer: Reverse primer:	TGA AGG AAC GGA CTG GCT TC GAG TGC ATA GGT GAG GAG GC
*Nurr1*	Forward primer: Reverse primer:	GAC CAG GAC CTG CTT TTT GA ACC CCA TTG CAA AAG ATG AG
*Lmx1a*	Forward primer: Reverse primer:	GAG ACC ACC TGC TTC TAC CG GCA CGC ATG ACA AAC TCA TT
*En1*	Forward primer: Reverse primer:	TCA CAG CAA CCC CTA GTG TG CGC TTG TCT TCC TTC TCG TT
*Pitx3*	Forward primer: Reverse primer:	CAT GGA GTT TGG GCT GCT TG CCT TCT CCG AGT CAC TGT GC
*Chl1*	Forward primer: Reverse primer:	TGG AAT TGC CAT TAT GTG GA CAC CTG CAC GTA TGA CTG CT
*Dat*	Forward primer: Reverse primer:	TTG CAG CTG GCA CAT CTA TC ATG CTG ACC ACG ACC ACA TA
*Drd2*	Forward primer: Reverse primer:	CTC AAC AAC ACA GAC CAG AAT GAA CGA GAC GAT GGA GGA
*Bdnf*	Forward primer: Reverse primer:	ACT ATG GTT ATT TCA TAC TTC GGT T CCA TTC ACG CTC TCC AGA

## Data Availability

All data supporting the stated results are available in the manuscript.
